# Tumor‐Regional Immune Microenvironment: A Critical Factor in the Design of Radiotherapy–Immunotherapy Combination Trials

**DOI:** 10.1002/ijc.70579

**Published:** 2026-06-09

**Authors:** Xuanwei Zhang, Hui Wang, Weiyan Tang, Kai Kang, Kateryna Onyshchenko, Taotao Huang, Dan Liu, Jianxin Xue, You Lu, Gabriele Niedermann, Ren Luo

**Affiliations:** ^1^ Division of Thoracic Tumor Multimodality Treatment, Cancer Center West China Hospital, Sichuan University Chengdu Sichuan China; ^2^ Department of Radiation Oncology, Cancer Center West China Hospital, Sichuan University Chengdu Sichuan China; ^3^ West China School of Medicine Sichuan University Chengdu Sichuan China; ^4^ Department of Radiation Oncology, Faculty of Medicine University of Freiburg Freiburg Germany; ^5^ Faculty of Biology University of Freiburg Freiburg Germany; ^6^ Laboratory of Biosynthesis of Nucleic Acids Institute of Molecular Biology and Genetics of NASU Kyiv Ukraine; ^7^ Department of Urology Emory University School of Medicine Atlanta Georgia USA; ^8^ Laboratory of Clinical Cell Therapy West China Hospital, Sichuan University Chengdu Sichuan China

**Keywords:** immune aggregates, immune checkpoint blockade, radiotherapy–immunotherapy, tumor‐draining lymph nodes, tumor‐regional immune microenvironment

## Abstract

Clinical trials combining radiotherapy (RT) with immune checkpoint blockade (ICB) have shown improved outcomes in only a fraction of patients, and optimal strategies for integrating these modalities remain under intense investigation. With a few exceptions, phase III combination trials have yielded disappointing results. This may be due to detrimental effects of RT on the tumor‐regional immune microenvironment (TRIME), including tumor‐draining lymph nodes (TDLNs) and intratumoral immune aggregates such as tertiary lymphoid structures. TDLNs are crucial for generating tumor‐specific T cells, including progenitors of exhausted T cells. Intratumoral immune aggregates are hubs for immune cell interaction participating in induction or reactivation of antitumor immune responses. Understanding the biological role of the TRIME in RT/ICB‐induced antitumor immunity is therefore essential for designing RT–immunotherapy combination trials. This review also highlights several potential strategies to optimize RT/ICB combination therapy. One approach involves modifying treatment schedules, for example, delaying RT to TDLNs until after ICB to allow effective immune priming. Another promising strategy is the integration of advanced imaging techniques into RT planning to improve precision and minimize radiation exposure to TDLNs. Applying unconventional RT with lower total dose or to selective areas may help preserve immune aggregates within tumors, potentially enhancing synergy with ICB. Another important approach is the use of dendritic cell agonists to boost the function of the TRIME. These approaches may help unlock the full therapeutic potential of RT/ICB combinations.

## Graphical abstract

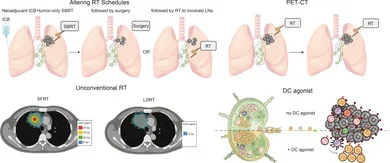

**Abbreviations**
APC: antigen‐presenting cell
ARG1: arginase 1
ATR: ataxia telangiectasia and Rad3‐related
CCL5: C–C chemokine ligand 5
CCR5: C–C chemokine receptor 5
CCR7: C–C chemokine receptor 7
cCRT: concurrent chemoradiotherapy
CD28: co‐stimulatory receptor on T cells
CD80/CD86: co‐stimulatory molecules on antigen‐presenting cells
cGAS: cyclic GMP–AMP synthase
cGAS–STING: cyclic GMP–AMP synthase–stimulator of interferon genes pathway
CRT: chemoradiotherapy
CTLA‐4: cytotoxic T‐lymphocyte‐associated protein 4
CXCL9/10/11: C–X–C chemokine ligands 9/10/11
CXCL16: C–X–C chemokine ligand 16
CXCR3: C–X–C chemokine receptor 3
CXCR6: C–X–C chemokine receptor 6
DAMPs: damage‐associated molecular patterns
DC: dendritic cell
dsDNA: double‐stranded DNA
ENI: elective nodal irradiation
FDA: U.S. Food and Drug Administration
FDG: fluorodeoxyglucose
Flt3L: Fms‐like tyrosine kinase 3 ligand
FTY720: Fingolimod
GM‐CSF: granulocyte‐macrophage colony‐stimulating factor
HEV: high endothelial venule
HMGB1: high‐mobility group box 1
HNSCC: head and neck squamous cell carcinoma
HPV: human papillomavirus
hRT: hypofractionated radiotherapy
ICB: immune checkpoint blockade
ICD: immunogenic cell death
IDO1: indoleamine 2,3‐dioxygenase 1
IFN: interferon
IFN‐I: type I interferon
IFN‐γ: interferon gamma
IFRT: involved‐field radiotherapy
IL: interleukin
IL‐2c: interleukin‐2 complex
iNOS: inducible nitric oxide synthase
LDRT: low‐dose radiotherapy
LNs: lymph nodes
MAVS: mitochondrial antiviral‐signaling protein
MDA5: melanoma differentiation‐associated protein 5
MDSCs: myeloid‐derived suppressor cell
MHC: major histocompatibility complex
MPR: major pathological response
NETs: neutrophil extracellular traps
NK: natural killer cell
NPC: nasopharyngeal carcinoma
NSCLC: non‐small‐cell lung cancer
OS: overall survival
pCR: pathological complete response
PD‐1: programmed cell death protein 1
PD‐L1: programmed death‐ligand 1
PET: positron emission tomography
PET‐CT: positron emission tomography‐computed tomography
PFS: progression‐free survival
PULSAR: personalized ultrafractionated stereotactic adaptive radiotherapy
RIG‐I: retinoic acid‐inducible gene I
RT: radiotherapy
S1P: sphingosine‐1‐phosphate
SBRT: stereotactic body radiotherapy
SFRT: spatially fractionated radiotherapy
STING: stimulator of interferon genes
TANs: tumor‐associated neutrophil
TDLN: tumor‐draining lymph node
Tef: effector T cell
Tex: terminally exhausted T cell
TIL: tumor‐infiltrating lymphocyte
TLR: toll‐like receptor
TLS: tertiary lymphoid structure
TME: tumor microenvironment
TMLN: tumor‐metastasized lymph node
Tpex: progenitor exhausted T cell
Treg: regulatory T cell
TREX1: three prime repair exonuclease 1
TRIME: tumor‐regional immune microenvironment
αCTLA‐4: anti‐CTLA‐4
αPD‐1: anti‐PD‐1

## 1 Introduction

Radiotherapy (RT) is a fundamental treatment modality used in more than 50% of cancer patients [1]. Conventional RT delivers 1.8–2.2 Gy per day over 5–7 weeks [2]. Advances in imaging and precision RT have enabled higher doses per fraction, known as hypofractionated RT (hRT). Moderate hRT delivers 2.5–6 Gy per fraction, while extreme hRT, or stereotactic body RT (SBRT), exceeds 6 Gy per fraction in ≤ 5 fractions [3]. Once thought to act solely via direct tumor cell killing, RT is now recognized to also elicit potent antitumor immune responses [4, 5, 6].

Immune checkpoint blockade (ICB) revolutionized cancer therapy with the 2011 U.S. Food and Drug Administration (FDA) approval of ipilimumab for melanoma [7, 8]. Initially, ICB was thought to act by reinvigorating tumor‐resident tumor‐infiltrating lymphocytes (TILs) [9], but later studies revealed that PD‐1/PD‐L1 blockade primarily drives a proliferative burst of progenitor exhausted CD8^+^ T cells (Tpex), which differentiate through effector‐like (Tef) to terminally exhausted (Tex) states [10]. As ICB response rates remain low (10%–25%) [8], combination approaches, particularly with RT, which enhances immune activation, are being actively investigated [5, 6].

Tumor‐draining lymph nodes (TDLNs) are pivotal to antitumor immunity, acting as key hubs for immune cell activation and coordination [11]. However, metastasis can disrupt TDLN function, converting them into tumor‐metastasized lymph nodes (TMLNs) that facilitate immune tolerance and systemic spread [11, 12]. These dual roles make TDLNs a complex target in cancer therapy [11, 12]. Alongside TDLNs, intratumoral immune aggregates such as tertiary lymphoid structures (TLS) act as local immune hubs within the tumor microenvironment (TME). Unlike encapsulated TDLNs, they are directly exposed to tumor‐derived stimuli, allowing dynamic interaction with the surrounding TME [13]. Recognizing their interconnected roles, we propose the concept of the tumor‐regional immune microenvironment (TRIME) to describe region‐specific immune determinants shaping antitumor responses.

This review discusses the role of TRIME in antitumor responses and the immunobiological rationale for TRIME sparing in the design of RT–immunotherapy combination trials, thereby bridging insights from bench to bedside.

## 2 Role of TDLNs and TMLNs in Antitumor Immunity

### 2.1 Antigen Capture and Transport by Dendritic Cells to TDLNs

**FIGURE 1:**
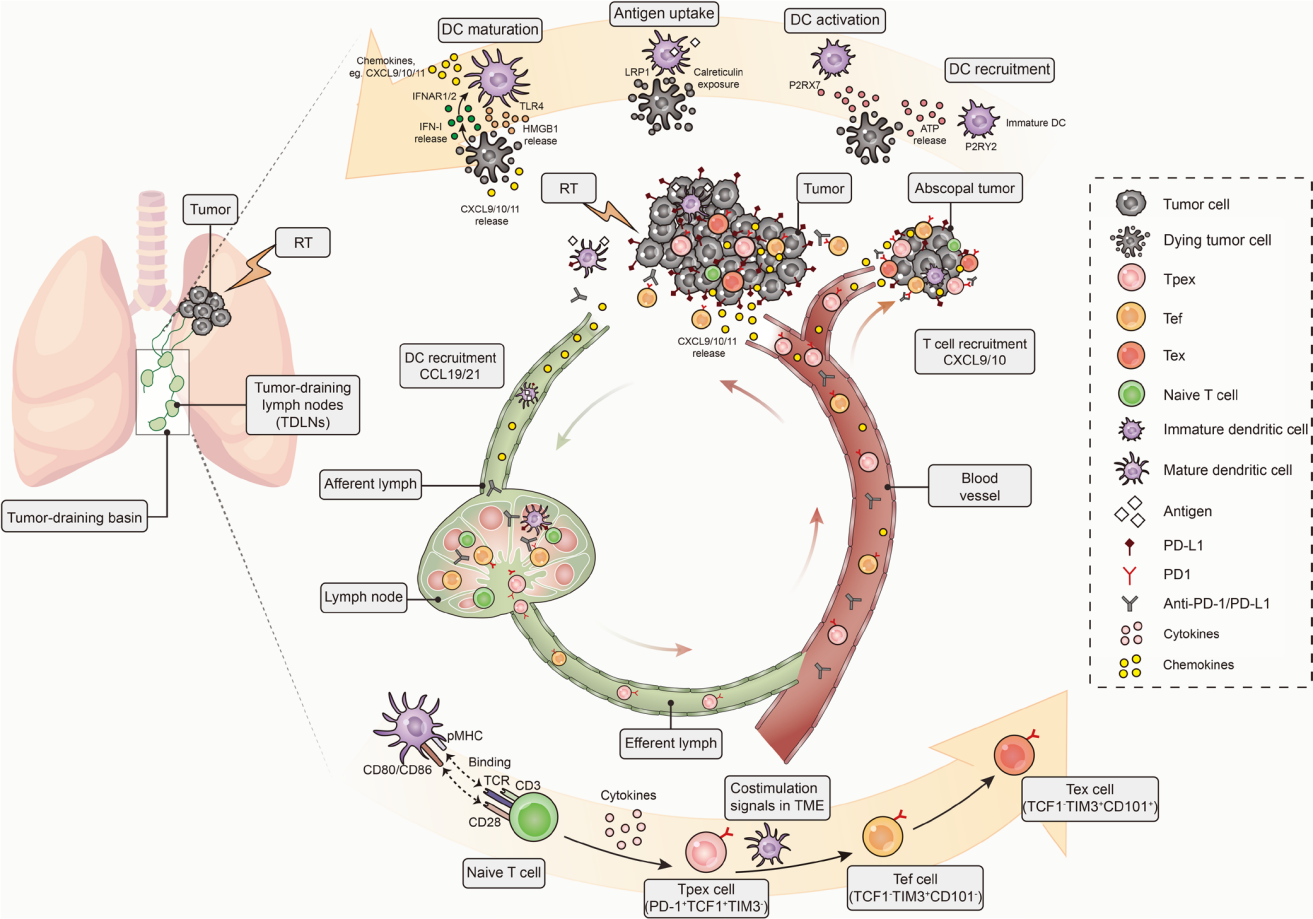
RT promotes DC maturation in the TME and T cell priming in TDLNs, while chemokines guide activated T cells to both irradiated and distant abscopal tumors. Localized RT starts immune responses by releasing tumor‐associated antigens and inducing immunogenic cell death (ICD). During ICD, damage‐associated molecular patterns (DAMPs) are released, which help recruit and activate dendritic cells (DCs). Dying tumor cells release ATP, which binds to the purinergic receptor P2Y2 (P2RY2) and directs DC precursors to the tumor site. Concurrently, ATP activates DCs through the purinergic receptor P2X7 (P2RX7) signaling pathway. For antigen uptake, surface‐exposed calreticulin enhances the phagocytosis of tumor antigens by engaging low‐density lipoprotein receptor‐related protein 1 (LRP1) on DCs. The binding of high‐mobility group box 1 (HMGB1) to Toll‐like receptor 4 (TLR4) promotes the DC maturation. Subsequently, mature DCs migrate to tumor‐draining lymph nodes (TDLNs) under the guidance of chemokines like CCL19 and CCL21. Within TDLNs, DCs deliver tumor‐derived antigens to naïve T cells via major histocompatibility complex (MHC) molecules, supported by co‐stimulatory signals like CD80/CD86 binding to CD28. This antigen presentation primes naïve T cells, promoting their differentiation into precursor exhausted T cells (Tpex; PD‐1^+^TCF1^+^TIM3^−^). These Tpex cells then traffic to the tumor microenvironment (TME), where they further differentiate into effector T cells (Tef; TCF1^−^TIM3^+^CD101^−^) and terminally exhausted T cells (Tex; TCF1^−^TIM3^+^CD101^+^). T cells are recruited to both primary and abscopal tumor sites through the bloodstream, which is facilitated by chemokines such as CXCL9 and CXCL10. Within the TME, T cell function can be suppressed by checkpoint molecules like PD‐1 and PD‐L1. However, treatment with immune checkpoint inhibitors (e.g., anti‐PD‐1/PD‐L1 antibodies) can block these interactions, thereby reinvigorating T cell function and enhancing antitumor immunity. This cascade emphasizes the crucial role of TDLNs in initiating adaptive immune responses and highlights the therapeutic potential of combining RT with immunotherapy to potentiate systemic antitumor immunity, effectively targeting both irradiated and non‐irradiated tumor sites.

Lymph nodes (LNs) serve as critical hubs for immune cell interactions, orchestrating innate and adaptive immunity by various processes. Immature dendritic Cells (DCs) within the tumor actively sample their environment [14] and become activated through toll‐like receptors (TLRs) or other pattern recognition receptors that engage with pathogen‐associated molecular patterns or damage‐associated molecular patterns (DAMPs), leading to CD80/CD86 upregulation and cytokine release [14, 15]. RT or chemotherapy can induce immunogenic cell death (ICD), which induces DAMPs such as surface‐exposed calreticulin, extracellular ATP, released high‐mobility group box 1 (HMGB1), and type I interferon (IFN) responses, thereby promoting DC activation and maturation, cross‐presentation of tumor antigens, and priming of CD8^+^ T cells (see Figure 1) [6]. Activated DCs upregulate CCR7 and migrate via lymphatics to TDLNs guided by CCL19/CCL21, while CCR7 likewise directs naïve T cells into TDLNs through high endothelial venules (HEVs) [16].

### 2.2 CD8 ^+^ T Cell Priming in TDLNs

In TDLNs, DCs present tumor antigens via major histocompatibility complex (MHC) molecules to naïve T cells. Antigen recognition (signal 1) initiates priming, while CD80/CD86–CD28 costimulation (signal 2) supports survival and proliferation. Cytokines from DCs provide signal 3, driving T cell effector differentiation (see Figure 1) [12, 14, 15].

### 2.3 T Cell Egress From TDLNs to the TME

Activated T cells exit LNs into the circulation, directed by the lipid sphingosine‐1‐phosphate (S1P) [14]. Activated T cells express CXCR3, which directs migration into tumors through its ligands CXCL9, CXCL10, and CXCL11, induced by IFNs, RT, or chemotherapy [17]. Within the TME, Tpex differentiate into Tef expressing high levels of cytotoxic molecules, such as perforin and granzymes [18]. This transition involves reduced CXCR3, promoting T‐cell retention in the TME, as demonstrated in our study [19]. Additional axes such as CCR5‐CCL5 and CXCR6‐CXCL16 also regulate T‐cell recruitment and localization in the TME (Figure 1) [20].

**FIGURE 2:**
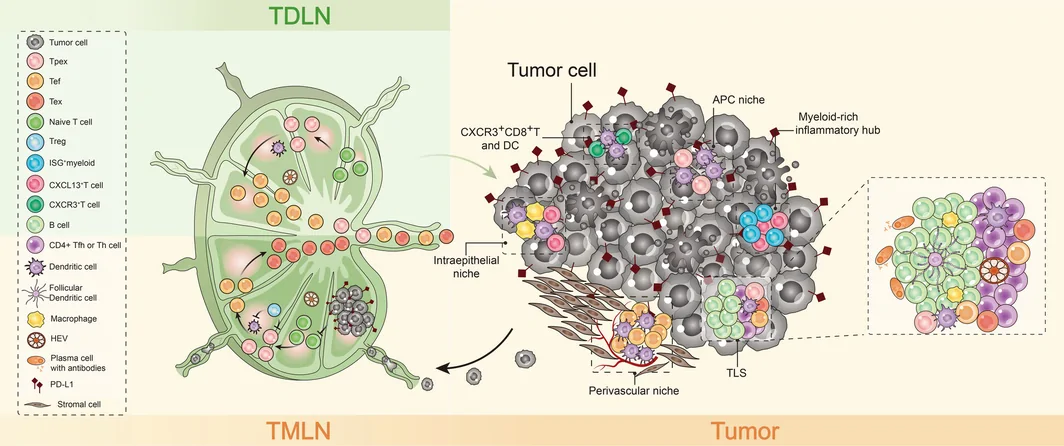
Tumor‐regional immune microenvironment: immune cell dynamics from TDLNs to immune aggregates in the TME. (Left) In tumor‐draining lymph nodes (TDLNs) and tumor‐metastasized lymph nodes (TMLN), naïve T cells are activated and differentiate into precursor exhausted T cells (Tpex) and effector T cells (Tef) through interactions with dendritic cells (DCs). However, in the context of nodal metastasis, regulatory T cells (Tregs) and tumor cells within TMLNs collectively promote the further differentiation of Tef cells into terminally exhausted T cells (Tex). (Right) Immune aggregates within the tumor microenvironment (TME): Tertiary lymphoid structures (TLS): Organized but unencapsulated lymphoid aggregates that develop in non‐lymphoid tissues under pathological conditions. TLS facilitate local antigen presentation and T‐cell–B‐cell interactions, contributing to chronic inflammation and cancer progression. Antigen‐presenting‐cell (APC) niche: Clusters of APCs, such as DCs, which present tumor antigens to T cells and promote the activation of tumor‐specific immune responses. DCs secreting CXCL9/10/11 recruit CXCR3^+^ CD8^+^ T cells. Intraepithelial niche: Populated by macrophages and DCs that provide CD28‐mediated co‐stimulatory signals to T cells, thereby enhancing T‐cell activation and effector function. Perivascular niche: Perivascular CCR7^+^ DCs recruit CXCR6^+^ Tef cells via the CXCL16‐CXCR6 chemokine axis, supporting localized anti‐tumor immunity. Myeloid‐rich inflammatory hub: Characterized by interferon‐stimulated gene‐positive myeloid cells that recruit and activate CXCL13^+^ CD8^+^ T cells, thereby sustaining an inflammatory milieu within the TME.

After entering the TME via tumor‐associated HEVs, T cells may localize within TLS or other immune niches (see Figure 2) [13, 16, 21, 22, 23]. TLS are organized lymphocyte aggregates resembling LNs, often containing B cells, T cells, and DCs [13, 22, 23]. In contrast to LNs, which develop at predetermined anatomical locations, TLS arise in non‐lymphoid tissues during chronic inflammation, such as in cancer [13, 22, 23, 24].

### 2.4 TMLN‐Driven Immunosuppression

Immunosuppression can arise in TMLNs, impairing both local and systemic tumor control [11, 25, 26, 27]. In TMLNs, reduced CCL21 expression restricts T‐cell entry through HEVs, and decreased IL‐7 further inhibits T‐cell proliferation (see Figure 2) [28, 29]. Chronic type I/II IFN signaling elevates MHC‐I and PD‐L1 on tumor cells, enabling escape from natural killer (NK) cell‐ and T‐cell‐mediated surveillance [25]. LN metastasis also expands tumor‐specific regulatory T cells (Tregs), promoting distant tumor spread [25]. In melanoma, recurrence‐free patients showed enhanced lymphocyte activation, whereas those with recurrence displayed T‐cell suppression with elevated CTLA‐4 and PD‐L1, especially in distal TMLN regions [26]. LN metastasis has also been found to actively drive systemic immunosuppression in cancer by establishing myeloid–cancer‐associated fibroblast niches with IFN‐γ signaling, which remodel LN architecture, promote Treg activation and T‐cell dysfunction, and extend immune suppression to tumor‐free LNs, thereby facilitating metastatic progression [30]. In colon cancer, distinct immune–stromal states have been identified, in which fibroblast‐driven subtypes with suppressed T‐ and B‐cell activity were associated with advanced disease, poor prognosis, and partial reversibility by ICB [31].

In head and neck squamous cell carcinoma (HNSCC), PD‐L1 blockade repositions Tpex near DCs, promoting effector differentiation, but this process is disrupted in TMLNs due to immunosuppressive niches enriched with PD‐L1^+^ DCs and Tregs, limiting immunotherapy efficacy [27]. In addition, because RT or CRT can upregulate PD‐L1 expression on HNSCC cells [32, 33, 34], this may further reinforce adaptive immune resistance and provide a rationale for combining PD‐1/PD‐L1 blockade with RT/CRT in HNSCC.

## 3 The Role of TRIME in ICB Therapy

### 3.1 The Role of TDLNs in ICB Therapy

TDLNs are essential for the efficacy of ICB. αCTLA‐4 antibodies block CTLA‐4 from competing with CD28 for CD80/CD86 binding on DCs, thereby enhancing T‐cell co‐stimulation within TDLNs [7, 8]. This is supported by HNSCC mouse models, where removal of TDLNs abolished αCTLA‐4 efficacy [35].

Similarly, PD‐1/PD‐L1 blockade depends on TDLNs, as their removal markedly reduces therapeutic efficacy in mouse tumor models [36, 37]. Upon PD‐1/PD‐L1 blockade, Tpex cells undergo a “proliferative burst,” expanding in number and serving as a crucial source of tumor‐specific cytotoxic T cells [38]. Of note, PD‐1/PD‐L1 interactions in TDLNs rather than within the TME have been shown to correlate with prognosis in melanoma patients [39]. Mechanistically, PD‐L1 inhibition alleviates suppressive signals on PD‐L1^+^ cDCs in TDLNs, resulting in the proliferation of Tpex cells and the initiation of robust antitumor immunity [39]. TDLNs also serve as a reservoir for these Tpex cells, supporting their maintenance and subsequent migration to the TME, where they replenish Tef and Tex cell populations [40, 41].

### 3.2 The Role of Immune Aggregates in ICB Therapy

Lacking a capsule, TLS are directly exposed to the TME, shaping local immune responses. TLS amplify antitumor immunity by supporting T‐ and B‐cell activation, clonal expansion, and differentiation (see Figure 2) [13, 24]. Mature TLS with germinal centers produce high‐affinity antibodies that promote tumor cell killing via macrophages and NK cells [24, 42, 43]. TLS also harbor PD‐1^high^ CD8^+^ T cells and Tpex cells, both critical for ICB efficacy [13, 23, 44, 45, 46]. Pre‐existing mature TLS are strongly associated with favorable responses to ICB and prolonged survival across cancers [13, 24, 47].

However, TLS function can also be suppressed by inhibitory immune signals in the TME. TLS may recruit immunosuppressive cell subsets, including regulatory T cells (Tregs) and myeloid‐derived suppressor cells (MDSCs), which limit antitumor immunity [48]. Within TLS, Tregs are primarily located in the T‐cell zones, where they suppress local CD4^+^ and CD8^+^ T cell proliferation and function via CTLA‐4‐mediated DC inhibition and CD39‐driven adenosine production. In addition, follicular regulatory T cells, a CXCR5^+^ FOXP3^+^ subset localized within B cell follicles, can inhibit Tfh cells and dampen B‐cell responses, thereby impairing TLS maturation. MDSCs can infiltrate or accumulate at the periphery of TLS, where they may impair T‐cell priming and effector functions by producing reactive oxygen species and nitric oxide and by depleting essential amino acids, such as cysteine and arginine, which are critical for T‐cell function [48, 49].

Specialized immune niches are also essential for sustaining Tpex cells and augmenting antitumor immunity [21, 22, 50, 51, 52, 53]. Examples include: (1) APC (CD11c^+^MHCII^+^) niches in renal, prostatic, and bladder cancers, which facilitate Tpex‐cell survival, with diminished APC niche numbers correlated with decreased CD8^+^ T‐cell infiltration and abbreviated progression‐free survival (PFS) (refer to Figure 2) [50]; (2) intraepithelial niches in ovarian carcinoma, containing PD‐L1^+^ DCs and macrophages that enhance tumor‐specific TIL responses to PD‐1 inhibition via CD28 costimulatory signaling [51]; and (3) stromal perivascular niches in melanoma, wherein CCR7^+^ DCs release CXCL16, attracting CXCR6^+^ Tef cells through tumor‐associated HEVs and transmitting IL‐15 signals that sustain CXCR6^+^ Tef cell survival, thereby maximizing their anti‐tumor efficacy [21]. Additional niches, such as lymphonets [52] and myeloid‐rich inflammatory hubs [53], further illustrate the spatial organization of intratumoral immune responses and their potential as therapeutic targets (Figure 2).

In addition to the lymphocyte‐rich aggregates that support immunity, neutrophil‐enriched aggregates or neutrophil extracellular traps (NETs) represent another type of cellular cluster within the TME that may modulate antitumor immunity after RT and ICB. In most murine tumor models, neutrophils infiltrate tumors within 12–48 h after RT and aggregate in necrotic regions [54]. They have been implicated in tumor progression and immunosuppression via upregulation of indoleamine 2,3‐dioxygenase 1 (IDO1) and arginase 1 (ARG1), which deplete tryptophan or L‐arginine and thereby impair CD8^+^ T‐cell proliferation and effector functions [55]. In addition, RT‐treated tumor cells release HMGB1, which mediates NET formation by neutrophils [56, 57]. In a murine bladder cancer model and in patients with muscle‐invasive bladder cancer, NETs were shown to form a barrier at the tumor/stroma interface after RT that restricts CD8^+^ T cell infiltration into the tumor cell‐rich areas [58]. Thus, while RT and ICB aim to boost adaptive immunity, RT‐induced NETosis may simultaneously establish an immunosuppressive niche that limits the efficacy of tumor‐infiltrating lymphocytes. However, several studies have identified a subset of tumor‐associated neutrophils (TANs) with APC‐like features that enhance antitumor T‐cell responses, accompanied by increased expression of costimulatory molecules such as CD86, OX40L, and 4‐1BBL [59]. Nevertheless, a predominance of N2‐like TANs within tumors and a low peripheral blood lymphocyte‐to‐neutrophil ratio are consistently associated with poorer survival. Although low‐dose RT (LDRT) can polarize macrophages toward an M1 phenotype [60], it remains an important open question whether RT can reprogram pro‐tumoral neutrophils toward an anti‐tumoral, APC‐like state.

## 4 The Role of TRIME in RT‐Induced Antitumor Immunity

### 4.1 RT‐Induced Antitumor Immunity

RT not only kills tumor cells but can also elicit immunogenic effects, with evidence since the 1970s linking RT efficacy to an intact immune system [61]. The term *abscopal*, introduced by Robin Mole in 1953, originally referred to radiation effects occurring at a distance from the irradiated volume [62]. Demaria et al. demonstrated that the abscopal antitumor effect requires a competent immune system and is enhanced by combining RT with CTLA‐4 blockade in a CD8^+^ T cell‐dependent manner [4, 63]. Clinically, abscopal responses have been associated with systemic immune activation, such as increased NY‐ESO‐1‐specific antibodies and IFNγ^+^ CD4^+^ T cells in melanoma patients treated with RT plus ipilimumab [64].

As outlined in Section 2.1, RT can induce ICD, leading to DAMP exposure/release and subsequent DC activation. Activated DCs then migrate to TDLNs, where cDCs prime naïve tumor‐specific T cells. In parallel, RT‐induced chemokines such as CXCL9 and CXCL10 promote effector T‐cell recruitment into the TME (Figure 1). Preclinical studies indicate that hRT (6–12 Gy × 3–5 fractions) is most immunogenic, whereas higher per‐fraction doses (> 12 Gy) strongly induce Three Prime Repair Exonuclease 1 (TREX1), a DNA exonuclease that degrades cytosolic dsDNA, thereby preventing activation of the cyclic GMP‐AMP synthase‐stimulator of IFN genes (cGAS‐STING) type I IFN pathway and dampening the abscopal effect [65, 66]. However, RT‐induced immune activation is not restricted to the cGAS‐STING axis. RNA‐sensing pathways, including the retinoic acid‐inducible gene I (RIG‐I)/melanoma differentiation‐associated protein 5 (MDA5)–mitochondrial antiviral‐signaling protein (MAVS) pathway, can also contribute to type I IFN induction, for example in the presence of ATR inhibition; tumor cell‐intrinsic RIG‐I signaling has also been implicated in the immunogenic effects of RT and its synergy with ICB [67, 68]. In addition, ZBP1 may further expand the RT‐induced nucleic acid‐sensing network by promoting immunogenic necroptotic signaling and enhancing cGAS‐STING–IFN‐I activation [69].

While the T cell‐mediated abscopal effect is often robust in immunogenic preclinical tumor models, clinical efficacy in metastatic disease remains limited. In contrast, combining chemoradiotherapy (CRT) with adjuvant ICB has yielded promising results in phase III trials for patients with locally advanced cancers [1, 5, 6, 70, 71, 72]. Notably, the PACIFIC trial showed that consolidation therapy with durvalumab following concurrent CRT (cCRT) significantly improved PFS and overall survival (OS) in patients with unresectable stage III non‐small‐cell lung cancer (NSCLC) [72]. This may be attributable, at least in part, to the ability of chemotherapeutic agents to act synergistically with RT, not only through cytotoxic cooperation but also via immune modulation. In particular, anthracyclines and oxaliplatin are well‐established inducers of ICD, as validated by in vivo vaccination assays, the gold standard for assessing tumor immunogenicity. However, platinum agents commonly used in NSCLC and HNSCC—cisplatin and carboplatin—have generally been considered weak or non‐ICD inducers, largely because they fail to induce calreticulin translocation to the tumor cell surface [73]. Notably, recent studies have taken a more nuanced view by demonstrating robust vaccination efficacy with cisplatin‐ or carboplatin‐treated tumor cells across multiple tumor models [74, 75].

Consistent with this, our studies showed that cisplatin induces necroptosis‐associated ICD, elicits CD8^+^ T cell‐dependent antitumor immunity, and, when combined with hRT, contributes to a significant abscopal effect via the necroptosis‐driven mtDNA–IFN‐I axis [17, 76]. Although concerns have been raised that combining hRT with chemotherapy and ICB may result in excessive toxicity and thereby limit clinical applicability, we developed a novel strategy integrating partial SBRT to intratumoral subvolumes, LDRT to the entire tumor, and chemoimmunotherapy for advanced bulky solid tumors. This approach showed promising clinical activity in an unpublished retrospective cohort of 39 patients, as well as in two phase I trials (NCT05615142, NCT06349837) and an ongoing randomized phase II trial involving thoracic and abdominal bulky tumors [77, 78].

LDRT (≤ 2 Gy per fraction, total < 10 Gy) can polarize tumor‐associated macrophages toward an inducible nitric oxide synthase iNOS^+^ M1 phenotype, thereby recruiting T cells. LDRT‐induced antitumor responses involve NK, CD4^+^, and CD8^+^ T cells [60, 79, 80, 81]. Using bilateral tumor models, our group [79] and others [80] showed that combining SBRT to the primary tumor with LDRT to the abscopal site, plus ICB, markedly enhances systemic antitumor immunity through CD8^+^, CD4^+^ T cells and CXCL10 induction [79, 80]. This strategy demonstrated safety and efficacy in our phase I clinical trial IHC‐001 (NCT03812549) [82].

Activated T cells express CXCR3, which directs migration into tumors through its ligands CXCL9, CXCL10, and CXCL11, which are induced by IFNs that can in turn be triggered by immunogenic RT or chemotherapy. Immune cell infiltration into tumors is a highly dynamic process that is strongly influenced by the radiation dose and fractionation schedule. Preclinical studies have identified a critical temporal window for immune recruitment following single‐dose or hypofractionated RT regimens delivered at higher doses per fraction. Antigen‐presenting cells (APCs) can already be clearly elevated by days 3–5 post‐RT [83, 84]. CD8^+^ cytotoxic T cells peak around days 5–8, followed by an increase in immunosuppressive CD4^+^ Tregs starting on days 8–10 [84, 85]. These kinetics suggest that spacing RT fractions to accommodate T‐cell priming and infiltration may enhance tumor control, particularly when combined with ICB, as suggested by findings in immunocompetent mouse tumor models [86, 87, 88]. One illustrative example is personalized ultrafractionated stereotactic adaptive radiotherapy (PULSAR), which clinically employs SBRT fractions with inter‐fraction intervals of a few weeks or even months [89]. Collectively, optimizing RT–immunotherapy synergy may require not only rational combinations with and timing of immunomodulatory therapies, as well as appropriate total and per‐fraction RT doses, but also immunobiologically informed spacing of RT fractions to accommodate phases of active T‐cell recruitment, thereby avoiding periods of immune suppression or dysfunction.

### 4.2 Importance of TDLNs in Radio(Immuno)Therapy

In 1972, Perez et al. showed that removing or irradiating TDLNs before or shortly after tumor implantation markedly reduced the curative effect of RT, which achieved an 83% cure rate in controls [90].

In the ICB era, Marciscano et al. found that including TDLNs in the RT field reduced tumor T‐cell infiltration, local response, and survival, regardless of CTLA‐4 blockade, though not with PD‐1 blockade [91]. Our group demonstrated that blocking T‐cell egress from TDLNs using FTY720 impaired both local and abscopal antitumor responses to hRT + αPD‐1 [92]. Similar results were reported by Liu et al. [93]. Compared with conventional RT (2 Gy × 8), hRT (9.8 Gy × 1) to the irradiated TDLN tends to reduce CD8^+^ T‐cell infiltration in the TME and decrease circulating CD8^+^ T‐cells [94].

Buchwald et al. found that a single 10 Gy hRT dose stimulated Tpex proliferation in TDLNs, while TDLN irradiation (3 Gy × 3) reduced the abscopal effect and Tpex cells in both irradiated and distant tumors, identifying TDLNs as key Tpex reservoirs [95]. Consistently, our recent work showed that IL‐2 complexes (IL2c) expanded hRT + αPD‐1‐induced tumor‐specific CD8^+^ T cells, enhancing both local and abscopal control; FTY720 experiments confirmed that TDLNs supplied these crucial Tpex cells, which expanded mainly in blood after IL2c treatment [19].

In abscopal mouse HNSCC models, elective neck irradiation (ENI) suppressed T‐cell expansion in TDLNs, reduced TME infiltration, and limited epitope spreading [96]. In contrast, tumor‐only SBRT enhanced systemic T‐cell memory but led to regional recurrence. Adding sentinel LN resection or RT after tumor‐only SBRT prevented regional spread while preserving immunity [96]. Likewise, adjuvant rather than neoadjuvant or concurrent TDLN irradiation maintained RT–immunotherapy efficacy by preserving the CCR7‐CCL19/CCL21 axis [97].

Collectively, these studies indicate that TDLN irradiation can impair radio(immuno)therapy efficacy, though not always tumor control [98]. For example, in mice, concurrent TDLN irradiation (6 Gy × 1) did not hinder local control and even improved outcomes when combined with tumor RT and αCTLA‐4, likely via IL‐7‐driven lymphopenia‐induced T‐cell expansion [98]. Clinically, targeted irradiation of metastatic LNs can still elicit abscopal responses [99]; our group reported a PD‐1‐resistant patient achieving complete regression of all irradiated and non‐irradiated lesions after SBRT to an affected LN with αPD‐1 [99]. Moreover, patients with resected stage III melanoma benefited from αCTLA‐4 despite LN removal, possibly through antigen transport to adjacent lymphosomes [100, 101].

LNs house naïve T cells that are highly radiosensitive, with ~50% undergoing apoptosis after 2 Gy exposure [102]. Their depletion contributes to RT‐induced lymphopenia [98]. Although pronounced lymphopenia did not impair local or abscopal control in preclinical studies [76, 98], in patients, severe or prolonged lymphopenia is often associated with poorer treatment response and survival [103]. Compared with T cells, B cells and NK cells exhibit a comparable degree of radiosensitivity; however, depending on the experimental context, they have been reported to be either more or less radiosensitive than T cells [104, 105, 106]. In contrast, monocytes are relatively radioresistant compared with T, B, and NK cells, and further differentiation along the monocyte lineage confers increased radioresistance, as observed in macrophages and DCs [107, 108]. Notably, macrophage viability and phagocytic function remain largely preserved following irradiation with single doses up to clinically relevant levels (≤ 2 Gy) [108, 109].

Relevant preclinical studies demonstrating the importance of TDLNs in radio(immuno)therapy are listed in Table 1.

**TABLE 1:** Preclinical studies demonstrated the importance of TDLNs in radiotherapy–immunotherapy combinations.

Tumor models	Treatment scheme	Efficacy	Mechanism	References
One‐tumor model (MC38‐OVA and B16‐OVA tumors)	Tumor RT (12 Gy × 1 f) + αCTLA‐4 ± ENI	ENI restrained RT‐mediated immune infiltration and diminished the efficacy of hRT + αCTLA‐4	ENI decreased the expression of CXCR3‐ and CCR5‐associated chemokines and CD8^+^ T‐cell trafficking	Marciscano et al. [91]
Abscopal (bilateral) B16‐CD133 tumor model	Primary tumor RT (9.18 Gy × 3 f) + αPD‐1 ± FTY720	FTY720 reduced local and abscopal antitumor effects induced by hRT + αPD‐1	FTY720 decreased tumor‐specific T cell infiltration into the TME	Zhang et al. [92]
Abscopal (bilateral) B16F10GP tumor model	Tumor RT (10 Gy × 1 f) ± ENI	ENI did not impair local tumor control but diminished the abscopal effect	ENI decreased the number of Tpex cells in irradiated and non‐irradiated tumor	Zachary et al. [95]
Abscopal (bilateral) MC38 or B16 tumor models	Primary tumor RT (12 Gy × 1 f) + αPD‐1 ± TDLN resection	Surgical removal of TDLNs restrained local and abscopal antitumor effects induced by hRT + αPD‐1	TDLN resection decreased CD8^+^ T cell infiltration and the M1/M2 macrophage ratio	Liu et al. [93]
Abscopal (bilateral) HNSCC tumor models	Tumor‐only SBRT (10 Gy × 1 f) ± concurrent or supplementing TDLN ablation (ENI or resection) post‐surgery	Concurrent TDLN ablation dampened the abscopal effect but supplementing TDLN resection or ENI prevented regional recurrence	Concurrent ENI decreased antigen‐specific T cells and epitope spreading while supplementing ENI preserved immune responses	Darragh et al. [96]
One‐tumor model (MC38 tumor)	Tumor RT + αCTLA4 ± concurrent ENI (6 Gy × 1)	Concurrent ENI did not impede local tumor control	A 6 Gy dose may not have completely eliminated T cells in the TDLNs, but instead led to lymphopenia‐induced proliferation, driven by increased physical space and elevated availability of IL‐7	Telarovic et al. [97]
Abscopal (bilateral) B16‐CD133 or C51 tumor models	Tumor RT (12 Gy × 2f to B16‐CD133 or 8 Gy × 2f to C51) + αPD‐1 + CD122‐targeted IL‐2c ± FTY720	FTY720 reduced local and abscopal antitumor effects induced by hRT + αPD‐1 + IL‐2c	FTY720 decreased tumor‐specific T cell infiltration into the TME	Onyshchenko et al. [19]
Murine model of LN metastasis	Tumor‐only SBRT (15 Gy × 1 f) ± induction, concurrent or delayed ENI post tumor‐only SBRT	Induction and concurrent ENI diminished the efficacy of RT + ICB while delayed ENI after tumor‐only SBRT preserved the efficacy	Delayed ENI avoided the disruption of the CCR7‐CCL19/CCL21 homing axis of CD8^+^ T cells to TDLNs	Telarovic et al. [98]

Abbreviations: ENI, elective nodal irradiation; f, fraction; HNSCC, head and neck squamous cell carcinoma; IL‐2c, IL‐2 complexes; RT, radiotherapy; SBRT, stereotactic body radiotherapy; TDLN, tumor‐draining lymph node; TME, tumor microenvironment.

### 4.3 Importance of Immune Aggregates in Radio(Immuno)Therapy

Although the function of TDLNs in radio(immuno)therapy is well‐established, the involvement of immune aggregates remains underexplored. Immune aggregates, predominantly consisting of B and T cells [22], are essential for the efficiency of ICB but are markedly susceptible to high‐dose RT. Consequently, high‐dose RT may impair these structures, thereby reducing the therapeutic synergy with ICB. Thus, LDRT may represent a more suitable combination partner.

## 5 Strategies for Adapting RT to ICB Considering the TRIME

Because locoregional LN metastases are common in HNSCC, surgical removal or irradiation of TDLNs remains part of standard treatment [110, 111, 112]. In the phase III Keynote‐048 trial, pembrolizumab, alone or with chemotherapy, outperformed cetuximab‐based therapy in recurrent or metastatic HNSCC [113]. However, in locally advanced HNSCC, concurrent ICB with CRT failed to improve outcomes in the phase III Javelin 100 and Keynote‐412 trials [114, 115]. One possible explanation is that high RT doses to TDLNs may have impaired tumor‐specific T‐cell priming.

Similarly, in NSCLC, trials combining ICB with concurrent CRT have shown no added benefit, and it is well conceivable that high RT doses to TDLNs are an important contributing factor. The phase III PACIFIC‐2 trial (durvalumab + cCRT) failed to improve PFS or OS compared with cCRT alone [116], the CheckMate 73 L trial (cCRT + nivolumab ± ipilimumab) likewise showed no PFS advantage over the PACIFIC regimen [117].

**FIGURE 3:**
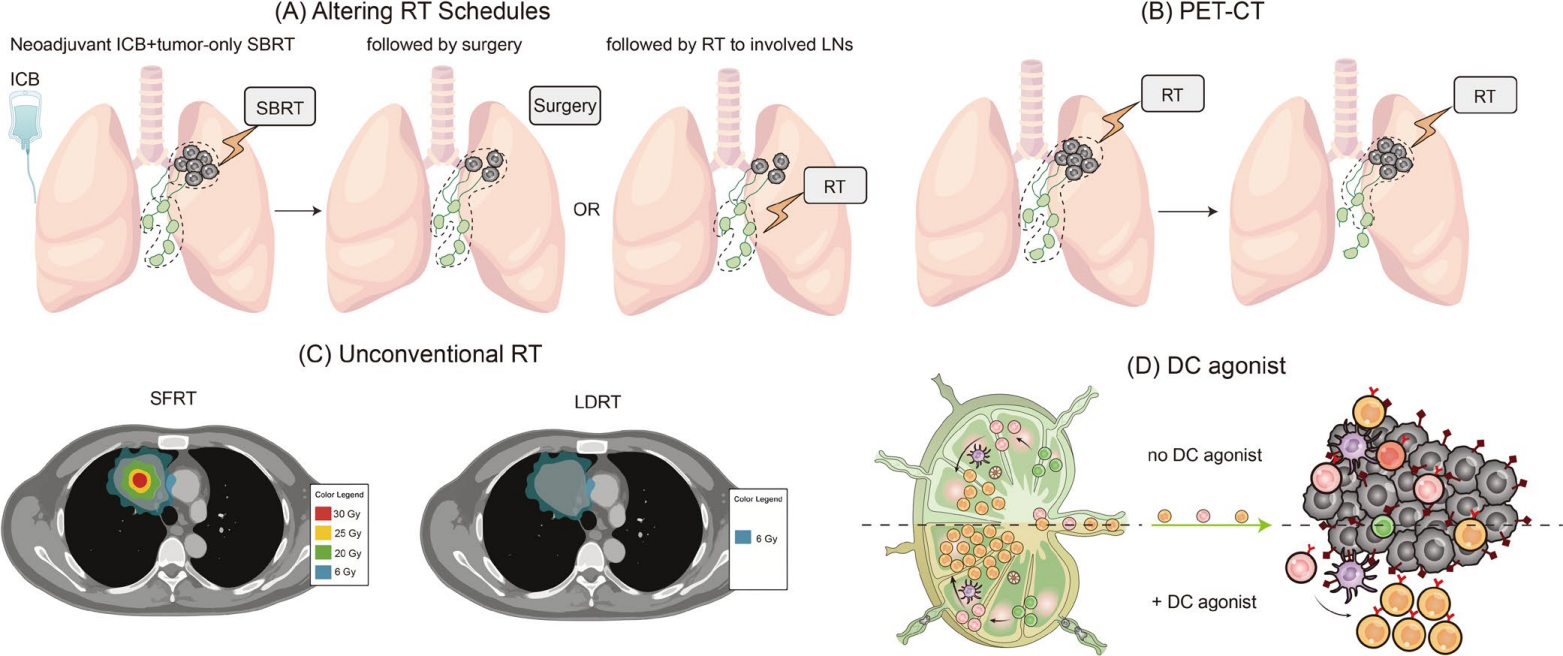
Strategies to adapt RT to ICB. (A) Optimization of radiotherapy (RT) scheduling: Employing neoadjuvant tumor‐targeted stereotactic body radiotherapy (SBRT) combined with immune checkpoint blockade (ICB), followed by either surgical resection or delayed irradiation of tumor‐draining lymph nodes (TDLNs) to preserve T‐cell priming. (B) Minimization of RT‐induced damage to the tumor‐regional immune microenvironment (TRIME) through PET‐CT‐guided treatment planning; this approach enables targeted tumor irradiation while reducing exposure of non‐involved TDLNs. (C) Preservation of intratumoral immune aggregates such as tertiary lymphoid structures (TLS) via spatially fractionated radiotherapy (SFRT) or low‐dose radiotherapy (LDRT). (D) Reprogramming of the TRIME to overcome immunosuppression by combining RT with immune‐modulatory agents such as GM‐CSF, Flt3L, TLR agonists, STING agonists, CD40 agonists, or T cell‐stimulating cytokines (e.g., IL‐2c) to enhance antigen presentation and promote T cell activation.

Hence, to achieve more personalized and precise lymphatic ablation for patients, it is essential to gain a deeper understanding of cancer biology, the interplay between tumors and the TRIME, and the anticancer immune response. Strategies for adapting RT to ICB, considering the TRIME, are shown in Figure 3.

### 5.1 Altering RT Treatment Schedules to Adapt to ICB

Clinical trials that attempted to adapt RT to ICB by altering treatment schedules are listed in Table 2 (see also Figure 3A).

**TABLE 2:** Clinical trials adapting RT to ICB.

Phase	Cancer type	No. of pts	TNM stage	Treatment scheme	RT dose	Endpoint	Safety	Efficacy	Clinical trials
Ib	Locally advanced HNSCC (primarily HPV+)	21	I–III for HPV+; III–IV for HPV−	Neoadjuvant tumor‐only SBRT with Nivolumab	24 Gy/3f	Unplanned delay in surgery	No DLTs and no treatment‐related surgical delays	MPR 86%, pCR 67%	Leidner et al. [118] (NCT03247712)
I/Ib	HPV‐negative locally advanced oral cavity HNSCC	21	II–IVb	Neoadjuvant SBRT tumor‐only with Durva	12–24 Gy/2–4f	MTD and DLTs	The most common grade 3–4 AE: oral mucositis (19%).	MPR 75%, pCR 44%	Darragh et al. [96] (NCT03635164)
Ib/II	HPV‐positive Oropharyngeal Cancer	19	T0‐3/N0‐2b	Neoadjuvant SBRT tumor‐only with Durva ± Tremelimumab	25 Gy/5f	Incidence of AE for the phase Ib and 2‐year PFS for the phase II	N/A	95% downstaged; 47% pCR	Ma et al. [119] (NCT03618134)
II	Early‐stage NSCLC	60	I–III	Durva versus Durva + neoadjuvant SBRT to primary tumor	24 Gy/3f	MPR	Grade 3–4 AE: 17% (Durva) versus 20% (Durva + SBRT)	MPR 53.3%/pCR 26.7% (SBRT + Durva) versus 6.7%/0% (Durva)	Altorki et al. [120] (NCT02904954)
II	Stage II–III, unresectable, locally advanced NSCLC	61	II–III	SBRT to primary tumor + standard RT to LNs + concurrent platinum doublets + adjuvant Durva	Primary tumor (50–54 Gy/3–5f); LNs (60 Gy/30f)	1‐year PFS	Serious AEs: 18%; Treatment‐related deaths: 7%.	1‐year PFS: 62.8%; mPFS: 25.3 months; mOS: 47.1 months.	Heinzerling et al. [121] (NCT03141359)
II	Stage IIA–IIIB NSCLC	46	IIA–IIIB	Neoadjuvant SBRT to the primary tumor + Platinum‐based chemo + Tislelizumab	24 Gy/3f	MPR	AE ≥ 3: 26%; 1 death from grade 5 chemo‐related neutropenia after first cycle	MPR 76%, pCR 52%	Zhao et al. [122] (NCT05319574)

Abbreviations: AE, adverse event; DLT, dose‐limiting toxicity; Durva, Durvalumab; HNSCC, head and neck squamous cell carcinoma; HPV, human papillomavirus; ICB, immune checkpoint blockade; LN, lymph node; MPR, major pathological response; MTD, maximum tolerated dose; NSCLC, non‐small cell lung cancer; OS, overall survival; pCR, pathological complete response; PFS, progression‐free survival; RT, radiotherapy; SBRT, stereotactic body radiotherapy.

#### 5.1.1 Neoadjuvant Tumor‐Only SBRT With ICB Followed by Radical Surgery

As shown in the preclinical studies discussed in Section 4.2 [96, 97], delaying TDLN irradiation until after ICB has exerted its effects may represent a rational treatment strategy. This is further supported by a recent preclinical study using a HNSCC model, which demonstrated that late resection of the TDLNs (6 days after ICB initiation) was more effective in controlling both primary tumors and LN metastases compared to early resection (1 day after ICB) [35].

In a phase Ib trial of 21 mostly HPV‐positive HNSCC patients, neoadjuvant SBRT (8 Gy × 3) plus nivolumab with TDLN sparing was well tolerated, achieving 86% major pathological response (MPR) and 67% pathological complete response (pCR), with 90% downstaging and allowing most patients to avoid adjuvant RT (Table 2) [118]. Another phase I/Ib trial of 21 patients with HPV‐negative, locally advanced HNSCC tested neoadjuvant SBRT (12–24 Gy in 6 Gy fractions) plus durvalumab [96]. Among those receiving 18–24 Gy, 75% achieved MPR and 44% pCR. Responders showed more CD103^+^CD39^+^CD8^+^ effector T cells, fewer Tregs, and greater T‐cell clonal expansion (Table 2) [123]. However, a phase Ib/II trial showed that neoadjuvant SBRT (5 Gy × 5) to the tumor with durvalumab ± tremelimumab was associated with early LN recurrence in HPV‐positive oropharyngeal cancer, even with concurrent or adjuvant ICB (Table 2) [119].

In a randomized phase II trial, neoadjuvant SBRT (8 Gy × 3) to the primary tumor only, combined with durvalumab, resulted in an MPR rate of 53% and a pCR rate of 27%, compared with 7% and 0%, respectively, for durvalumab alone, with a shorter time to surgery (5.3 vs. 12–16 weeks) and no added toxicity [120]. Inspired by the immunogenicity and safety of SBRT with ICB in this trial [120], integrating TDLN‐sparing SBRT with standard neoadjuvant chemoimmunotherapy may be a promising next step. In the single‐arm phase II SACTION0 trial, neoadjuvant SBRT to the primary tumor (8 Gy × 3), followed by tislelizumab plus chemotherapy, achieved an MPR rate of 76% and a pCR rate of 52% (Table 2) [122]. These outcomes suggest a potential improvement in MPR and pCR compared to historical rates (MPRs, 30%–56%; pCRs, 17%–41%) in neoadjuvant chemoimmunotherapy trials [122, 124, 125, 126].

#### 5.1.2 Tumor‐Only SBRT With ICB Followed by Standard Chemoimmunoradiotherapy

A phase 2 trial evaluated the addition of tumor‐only SBRT to standard chemoimmunoradiotherapy (PACIFIC regimen) in 61 patients with unresectable, locally advanced NSCLC (Table 2) [121]. Patients received SBRT to the primary tumor (50–54 Gy in 3–5 fractions), followed by conventional RT to involved LNs (60 Gy in 30 fractions) with cCRT [121]. Consolidation durvalumab was administered to patients without disease progression post‐cCRT. One‐year PFS was 62.7% overall and 69.6% in PACIFIC‐eligible patients, higher than the 55.7% reported in PACIFIC although the study did not meet its prespecified primary endpoint (> 60%) [121, 127]. The regimen showed favorable efficacy and fewer toxicities, supporting the ongoing phase III NRG LU008 trial [128]. These findings suggest a strategy of delivering ICB concurrently with or shortly after tumor‐only SBRT, followed by standard chemoimmunoradiotherapy. This approach preserves TDLNs during SBRT plus ICB until cCRT, potentially enhancing immune activation and treatment efficacy.

In the neoadjuvant SACTION0 trial, SBRT plus two cycles of chemoimmunotherapy achieved a 76% LN clearance rate (Table 2) [122]. Thus, administering tumor‐only SBRT/ICB/chemotherapy before the PACIFIC regimen may shrink LN targets, reduce RT toxicity, and enhance antitumor immunity.

### 5.2 Minimizing RT Exposure of the TRIME

#### 5.2.1 Minimizing of RT Dose to TDLNs

In nasopharyngeal carcinoma (NPC), reducing LN irradiation can lessen toxicity without compromising efficacy. A phase III trial showed that elective upper‐neck irradiation achieved similar 3‐year regional control to whole‐neck RT, with fewer late toxicities [129]. Another study found that sparing medial retropharyngeal LNs was non‐inferior for local control and reduced dysphagia [130].

Unlike in NPC, reducing LN target volumes in HNSCC remains under investigation. A phase II trial evaluated de‐escalation from elective neck irradiation to involved‐field RT (IFRT, targeting only radiographically/clinically involved gross LNs) after induction chemotherapy plus nivolumab [131]. Among 36 unresectable, HPV‐negative patients, those achieving ≥ 50% tumor shrinkage received 66 Gy to involved LNs, whereas others received standard 70–75 Gy. Two‐year PFS and OS matched historical cCRT outcomes, with fewer treatment‐related toxicities.

In locally advanced NSCLC, the omission of elective nodal irradiation (ENI) has long been standard, with locoregional control comparable to approaches that include ENI [132, 133]. In RTOG 0515, ^18^F‐FDG‐PET‐CT guided IFRT reduced target volumes and improved dose distribution, with only 2% nodal failures outside the treated fields [132]. The PET‐Plan trial further confirmed that PET‐based planning without mediastinal ENI achieved non‐inferior locoregional control, supporting elimination of mediastinal ENI [133].

Despite its advantages, PET‐CT‐guided planning is not widely used. PET‐CT‐guided planning to limit TDLN irradiation may enhance RT/ICB synergy (Figure 3B).

#### 5.2.2 Unconventional RT Techniques to Reduce Dose to Immune Aggregates

TLS and immune niches in the TME are vital for antitumor immunity [13, 23] but are highly radiosensitive. High‐dose RT can easily kill B and T cells in aggregates and damage HEVs via endothelial apoptosis [134]. In mice, hRT (11.7 Gy × 1) caused transient TLS loss, with recovery after 14 days [135]. Similarly, in brain metastases, preoperative stereotactic radiosurgery (12–27 Gy) was associated with an initial decrease in CD8^+^ T cells, followed by rebound by day 6; higher APC niche density correlated with improved local control [136]. Rather than damaging immune aggregates, LDRT (1 or 2 Gy in a single fraction) promoted early TLS formation, and combining LDRT with αPD‐1 therapy further enhanced TLS development and antitumor responses in mice [137].

Spatially fractionated radiotherapy (SFRT) is a nonconventional RT approach that delivers a non‐uniform pattern of high‐dose “peaks” and low‐dose “valleys” within the same treatment field. It was originally developed to treat bulky tumors by delivering high local doses while limiting toxicity to surrounding normal tissues. SFRT is currently being explored in preclinical and clinical settings across multiple tumor types, including sarcomas, HNSCC, NSCLC, and pancreatic cancer. SFRT may be a promising partner for immunotherapy: high‐dose regions can induce ICD, while low‐dose regions may help preserve immune aggregates. This spatial separation could be optimized empirically [77, 138] or guided by advanced immunoimaging to precisely guide high‐dose RT away from immune‐rich areas. The combination of heterogeneous RT and ICB has been explored in both preclinical and clinical settings by our group [77] and others [138, 139, 140].

Neoadjuvant chemoimmunotherapy is the current standard for resectable NSCLC [126]; however, further improvements are needed to enhance therapeutic efficacy. RT, as a potent inducer of ICD, may present a promising partner if delivered with low toxicity. In this context, unconventional RT may offer unique immunomodulatory benefits. Incorporating these techniques into the neoadjuvant setting could augment the effects of chemoimmunotherapy and improve clinical outcomes (see Figure 3C).

### 5.3 Overcoming Immunosuppression in the TRIME

Numerous pharmacological strategies can be envisioned to overcome TRIME‐associated immunosuppression and enhance ICB (see also Figure 3D). One approach is to use cytokines such as granulocyte‐macrophage colony‐stimulating factor (GM‐CSF) and Fms‐like tyrosine kinase 3 ligand (Flt3L) to promote DC activation, differentiation, and migration [15, 141, 142]. Recently, Flt3L was reported to expand cDC1s that prime and sustain stem‐like CD8^+^ T cells in TDLNs, thereby broadening clonal T‐cell responses and enhancing αCTLA‐4 efficacy [143].

Another key strategy is the use of immunostimulatory adjuvants targeting TLRs. The cGAS‐STING pathway also plays a central role by inducing type I IFNs and cytokines that enhance DC maturation and CD8^+^ T‐cell priming. cGAS‐STING agonists are under intense investigation in clinical trials [144, 145].

Directly targeting DCs with antibodies, particularly αCD40 antibodies, is an emerging strategy in cancer immunotherapy. CD40 signaling licenses DCs to prime and activate cytotoxic T cells [146]. Early CD40 agonists showed limited efficacy due to dose‐limiting toxicities and suboptimal dosing, but next‐generation Fc‐engineered antibodies now offer improved potency and safety [147].

Leveraging T cell‐stimulating cytokines may also represent a promising strategy to overcome TDLN‐associated immunosuppression. In a mouse model of spontaneous lung adenocarcinoma, T‐cell dysfunction emerged early in mediastinal TDLNs, with Treg‐mediated impairment of DC function through suppression of co‐stimulatory molecule expression and cytokine production [148, 149]. Notably, administration of IL‐2 and IL‐12 during the priming phase prevented T cell dysfunction and significantly extended survival in tumor‐bearing mice [148]. We also reported that CD122‐directed IL2c expanded Tpex cells by > 50‐fold, enhancing the RT/αPD‐1‐induced abscopal effect [19].

## 6 Conclusions

In summary, preclinical studies indicate that the TRIME is critical for the efficacy of ICB, RT, and their combination. TDLNs, which are rich in radiosensitive naïve lymphocytes, are key sites for DC‐mediated T‐cell priming, and immune aggregates likewise support antitumor immunity. To maximize RT/ICB efficacy, uninvolved TDLNs and immune aggregates should be spared whenever feasible, while balancing this against the need to control LN metastases. One potential practical strategy is to modify clinical trial protocols so that TDLN ablation occurs after ICB, thereby preserving priming capacity while effectively eliminating LN metastases. In addition, advanced imaging (e.g., ^18^F‐FDG‐PET) can improve target volume delineation and may facilitate sparing of uninvolved TDLNs [133]. Finally, nonconventional RT, approaches such as LDRT or SFRT—particularly in the neoadjuvant setting—may help preserve immune aggregates within the TRIME and thereby enhance responses in combination with ICB.

In clinical practice, however, irradiation or surgical removal of multiple locoregional LNs levels is often unavoidable. This raises an important question: can remaining or more distal LNs functionally compensate for proximal TDLNs? Emerging evidence suggests this may occur in certain settings [101]. How best to boost the remaining LNs—for example, with DC agonists—is another important question worthy of further investigation. Beyond classical bench‐to‐bedside translation, bedside‐to‐bench research is also needed to refine our understanding of how the TRIME can be protected during combined RT and immunotherapy. In addition, computational approaches for predicting lymphatic progression patterns and data‐driven resources such as LyProX may help guide future treatment strategies [150, 151].

## Author Contributions

**Xuanwei Zhang:** conceptualization, investigation, funding acquisition, writing – original draft, writing – review and editing, supervision. **Hui Wang:** conceptualization, investigation, writing – original draft, methodology, visualization, writing – review and editing. **Weiyan Tang:** conceptualization, investigation, writing – review and editing. **Kai Kang:** writing – original draft, methodology, writing – review and editing. **Kateryna Onyshchenko:** conceptualization, writing – original draft, methodology, writing – review and editing. **Taotao Huang:** writing – review and editing, methodology. **Dan Liu:** writing – review and editing, methodology, visualization. **Jianxin Xue:** methodology, visualization, writing – review and editing, conceptualization. **You Lu:** conceptualization, investigation, funding acquisition, writing – review and editing, writing – original draft, methodology. **Gabriele Niedermann:** conceptualization, investigation, funding acquisition, writing – original draft, writing – review and editing, supervision, visualization. **Ren Luo:** conceptualization, investigation, methodology, software, funding acquisition, writing – original draft, writing – review and editing, visualization, project administration, supervision, resources, data curation.

## Funding

This work was supported by the Federal Ministry of Education and Research of Germany, 02NUK064C, West China Hospital, Sichuan University, HXQMX0054, Sichuan University, 2023SCUH0045, Natural Science Foundation of Sichuan Province, 2024NFSC1912, 2026NSFSC0652, National Natural Science Foundation of China, 82303694, 82350128.

## Conflicts of Interest

Y. Lu reports roles as invited speaker/project lead/principal investigator with Roche, AstraZeneca, BeiGene, and Hengrui Pharmaceuticals, and invited speaker role with Pfizer, Merck Sharp & Dohme, and Bristol‐Myers Squibb. The other authors declare no conflicts of interest.
